# Implant Supported Fixed Dental Prostheses Using a New Monotype Zirconia Implant—A Case Report

**DOI:** 10.3390/dj3030079

**Published:** 2015-09-17

**Authors:** Stefan Roehling, Georges Ghazal, Thomas Borer, Florian Thieringer, Michael Gahlert

**Affiliations:** 1 Clinic for Oral- and Cranio-Maxillofacial Surgery, Hightech Research Center, University Hospital Basel, University of Basel, Spitalstr. 21, 4031 Basel, Switzerland; E-Mails: florian.thieringer@usb.ch (F.T.); m.gahlert@knihagahlert.de (M.G.); 2 Clinic for Oral- and Cranio-Maxillofacial Surgery, Kantonsspital Aarau, Tellstr., 5001 Aarau, Switzerland; E-Mails: georges.ghazal@ksa.ch (G.G.); moser_borer@yahoo.de (T.B.); 3 Private Dental Clinic Doctors Moser and Borer, Missionsstr. 1, 4055 Basel, Switzerland; 4 Private Dental Clinic Doctors Kniha and Gahlert, Theatinerstr. 1, 80333 Munich, Germany

**Keywords:** zirconium dioxide, zirconia, osseointegration, monotype ceramic implants, case report, clinical application

## Abstract

Currently, titanium or specific titanium alloys are the most often used materials for the fabrication of dental implants. Many studies have confirmed the osseointegrative capacity and clinical long-term performance of moderately rough titanium implants. However, disadvantages have also been reported with regard to peri-implant infections and the titanium metal properties. Tooth colored ceramic implants have attracted the interest of clinicians since the end of the 1960s. Initially, alumina was used for the fabrication of ceramic implants; however, due to the poor biomechanical properties, alumina implants are not commercially available any more. Since end of the 1990s, zirconia has been established in dentistry due to its superior biomechanical properties compared to other oxide ceramics such as alumina. Currently, zirconia is the material of choice for the fabrication of ceramic implants. Zirconia implants show superior biocompatibility compared to titanium and other metals. Additionally, it has been reported that zirconia implants with a micro-rough surface topography show at least a comparable osseointegrative capacity and similar clinical survival rates to moderately rough titanium implants. The present case reports a fixed implant-supported reconstruction of a large edentulous space with compromised local bone conditions using new monotype zirconia dental implants with a micro-rough surface topography.

## 1. Introduction

Tooth replacement with dental implants on partially or fully edentulous patients has become a science-based treatment modality in dentistry. It relies on the structural and functional stabilization of the implant in the surrounding bone tissue, called osseointegration [1]. Currently, the most often used material for the fabrication of dental implants is titanium or a specific titanium–zirconium alloy [2]. Many studies have confirmed the osseointegrative capacity and the clinical long-term performance of titanium implants with a moderately rough surface topography [2]. Clinically, survival and success rates of more than 95% after follow-up periods of up to and after 10 years have been reported [3,4,5]. However, disadvantages with regard to the material properties of the metal titanium have also been reported. For example, unsatisfying aesthetical long-term results when the grey titanium color of the implant or abutment has become visible as a result of thin peri-implant soft tissue conditions or peri-implant crestal bone resorptions [6]. In addition to that, several unwanted chemical–biological interactions of titanium with the surrounding soft and hard tissues have been reported [7]. Animal experiments have shown increased titanium concentrations in the direct vicinity of titanium fiber felt implants [8] and in regional lymph nodes after placing of Titanium-Plasma-Sprayed (TPS) implants [9]. Even sensitizations and positive allergic tests of single patients towards titanium have been reported [10]. However, the clinical relevance of these biological findings/disadvantages is controversially discussed.

Due to its tooth like color, ceramic implants have already attracted interests of clinicians since end of the 1960s. At this time, mono- and polycrystalline alumina (Al_2_O_3_) was used as material for the fabrication of commercially available dental implants, for example the “Crystaline Bone Screw” (CBS), the well-known “Tübingen-Immediat Implant” or the “Bioceram dental implant” [11,12,13]. Many experimental studies showed that alumina implants could directly integrate into osseous host tissue [14,15]. In addition to that, several clinical studies investigated the clinical long-term performance of alumina dental implants and reported survival rates between 44.2% and 96.3% [16,17]. However, due to its poor biochemical properties (high level of hardness, high elasticity module, low level of resistance to bending and fracture strength), alumina was prone to fracture when implants were loaded extra axially [18]. Thus, currently alumina implant systems are not commercially available any more.

In the beginning of the 1990s, zirconium dioxide (zirconia, ZrO_2_) has been introduced to dentistry. In comparison to other oxide ceramics (such as alumina), zirconia shows superior biomechanical properties (high level of resistance to bending and fracture strength, low elasticity module) [19]. Laboratory investigations have shown that zirconia dental implants have the ability to withstand occlusal forces [20,21]. Thus, since the beginning of the 2000s, zirconia has been used as material for the fabrication of dental implants. Currently, two-piece (implant body and abutment as two individual elements) as well as one-piece (monotype, the abutment as an inherent part of the implant body) zirconia implants are commercially available. Besides geometry, the different zirconia implant systems also vary in their fabrication process and their surface topography.

With regard to the osseous integration, histological results from experimental studies have shown that zirconia dental implants can directly integrate into osseous host tissue under non-loaded and under loaded conditions [22,23,24,25]. Similar to titanium, it has been demonstrated that an increased surface micro-roughness correlates with an increased osseointegrative capacity [26,27]. In addition to that, it has been reported that zirconia implants with a micro-roughened surface topography show at least a comparable osseointegrative capacity to moderately rough titanium implants [28,29,30,31].

With regard to the clinical application of zirconia dental implants, currently only very little evidence-based data are available. A recently published literature review including studies that were published between 2006 and 2011 concluded that most of the clinical studies that have been conducted so far showed considerable shortcomings and only low levels of evidence [32]. Thus, from an evidence-based point of view, it seems to be quite reasonable that many practitioners are still very skeptical with regard to the clinical application of zirconia dental implants. However, it must be noted that the authors of the latter review did not include current clinical findings of zirconia dental implants of the latest generation with a micro-rough surface topography that report similar survival rates compared to moderately rough titanium implants [33]. In addition to that, many experienced dental implantologists are already convinced of the clinical advantages of ceramic implants. The present case reports a fixed implant-supported reconstruction of a large edentulous area with compromised local bone conditions in the esthetically highly relevant frontal area of the maxilla using a new monotype zirconia dental implant system with a micro-rough surface topography.

## 2. Case Report

**Figure 1:**
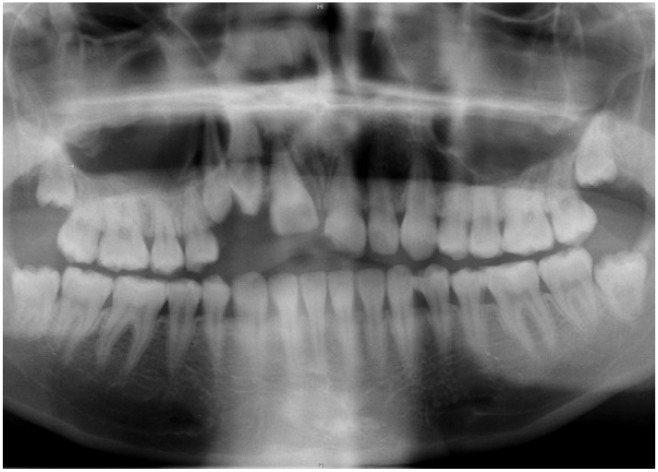
Panoramic radiographic investigation directly after trauma (Image: Institute of Radiology, Kantonsspital Aarau, AG, Aarau, Switzerland).

In February 2012, a 21 years old female patient was referred to our clinic on an emergency basis because she collapsed—as the result of orthostatism—and hit her face against the lavatory. The patient’s medical and dental history was completely unremarkable and she did not regularly take any medication and denied alcohol or tobacco abuse. The clinical examination showed extraorally a perforating laceration of the lower lip and intraorally, a severe plurifragmentary fracture of the alveolar process between teeth 11 and 14 (all teeth positions are reported according to the World Health Organization (WHO) classification). Teeth 14 to 21 were orally dislocated and additionally, tooth 21 showed an uncomplicated crown fracture without opening of the dental pulp. The radiographic investigation confirmed the severe fracture of the alveolar process (Figure 1).

As immediate measure, extraorally, the laceration of the lower lip was sutured. Intraorally, small bone and teeth fragments were removed and the soft tissue wound was cleaned and sutured. Due to the severe plurifragmentary fracture of the alveolar process, teeth 11 to 14 had to be extracted. Tooth 21 was repositioned and stabilized with a titanium splint. After three weeks of uncomplicated wound healing, the titanium splint was removed and a removable temporary prosthetic reconstruction was prepared. The clinical investigation at this time point already showed an osseous defect in region of teeth 11 to 13. Tooth 21 was clinically stable, showed a positive pulp vitality reaction and was temporarily reconstructed with a composite filling. In addition to that, the patient received a removable temporary prosthesis to replace the missing teeth 11 to 14.

Due to the patient’s age and the naturally healthy neighboring teeth in the maxilla, it was decided to initiate further diagnostic procedures to plan an implant-supported fixed prosthetic reconstruction.

**Figure 2:**
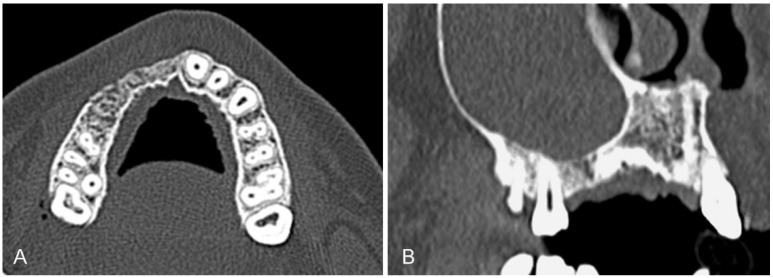
Dental CT-Scan at four months after trauma. Axial (**A**) and sagittal (**B**) CT-images (Images: Institute of Radiology, Kantonsspital Aarau, AG, Aarau, Switzerland).

Following that, in June 2012 after four months of osseous healing, a dental CT (Toshiba Aquilion CXL, Toshiba Medical Systems AG, Oetwil am See, CH; DLP (mGycm): 161.60) was performed. The three-dimensional radiographic investigation confirmed the clinical examination and showed a vertical bone defect of the labial and palatal wall of the alveolar process in region of teeth 11 to 14. Additionally, the CT scan showed large extensions of the maxillary sinuses resulting in a reduced horizontal bone height in region of tooth 15 and a maxillary sinusitis (Figure 2).

Consequently, the patient was informed that, prior to implant placement, a bone augmentation procedure with an autogenous bone block had to be performed. In addition to that, she was told that the implant placement in region of tooth 14 had to be combined with a simultaneous sinus floor elevation. Because of the maxillary sinusitis, the patient immediately received an oral antibiotic treatment with amoxicillin and clavulanic acid and decongestant nose drops for eight days according to in-house protocol.

**Figure 3:**
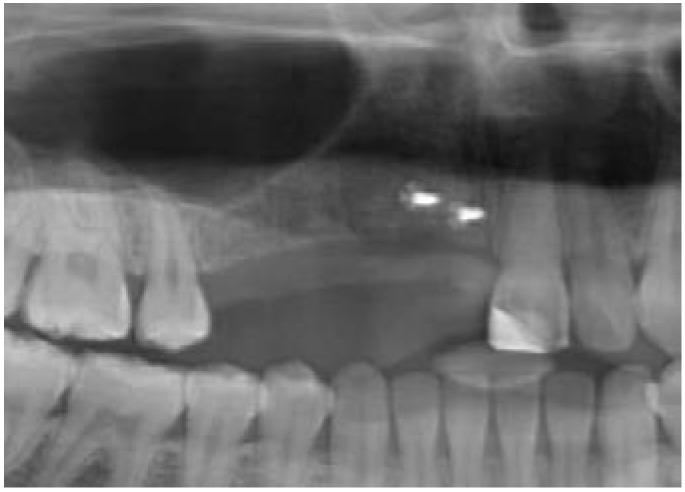
Panoramic radiographic investigation after fixation of the bone block. Surgeons: Dr. Stefan Roehling, Dr. Georges Ghazal (Image: Institute of Radiology, Kantonsspital Aarau, AG, Aarau, Switzerland).

In February 2013, an autogenous bone block (dimensions 10 × 20 mm) was harvested from the left mandibular ramus, transferred to the labial side of the maxilla in region of teeth 11 to 13 and fixed with two titanium osteosynthesis screws (Figure 3). To prevent soft tissue ingrowth, the block was covered with a collagen membrane (Bio Gide^®^, Geistlich Biomaterials, Baden-Baden, Germany). The surgery was performed in local anesthesia. Postoperatively, the patient received an oral antibiotic treatment with amoxicillin and clavulanic acid for eight days according to in-house protocol.

**Figure 4:**
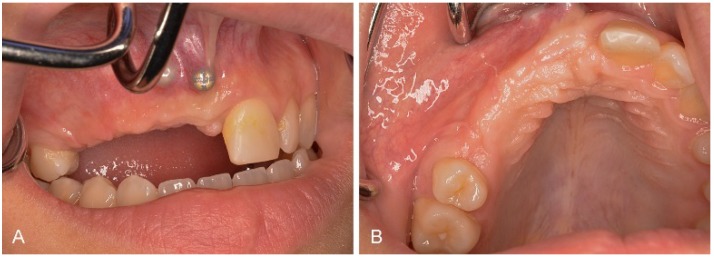
Clinical situation at six months after autogenous bone augmentation.

After six months of uneventful osseous healing, the clinical examination showed completely non-irritated soft tissue conditions and acceptable, sufficient bone volume; however, especially the palatal side still showed some small vertical bone deficiencies (Figure 4). Following this, the planning of the prosthetic reconstruction was initiated.

**Figure 5:**
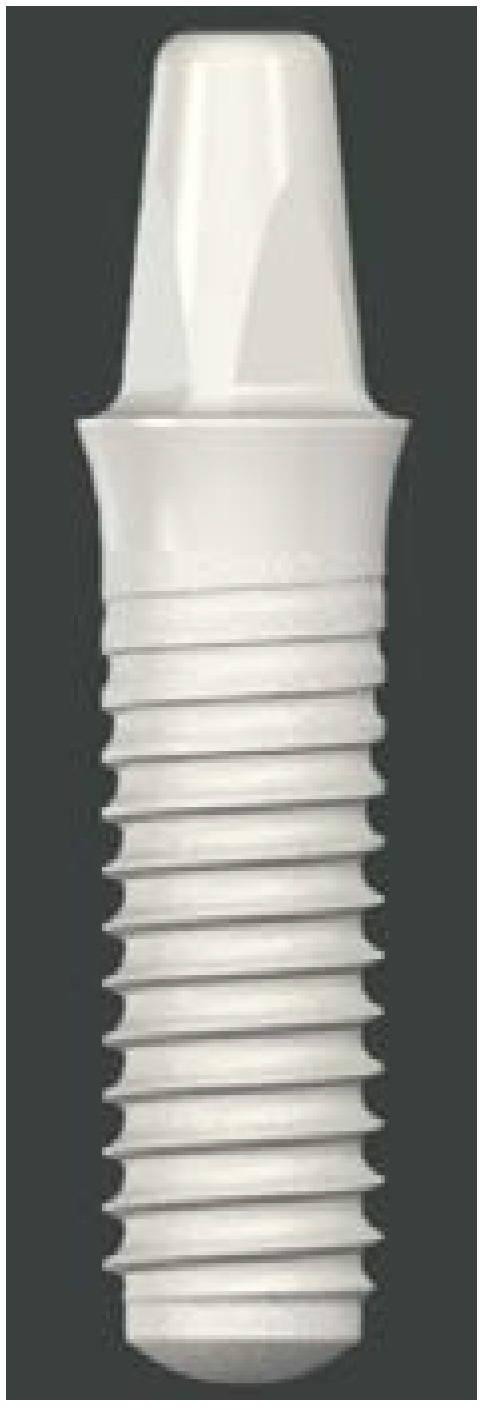
Monotype full ceramic ZrO_2_ implant (Straumann^®^ PURE Ceramic Implant, Institut Straumann AG, Basel, Switzerland).

For the implant-supported reconstruction, it was decided to use a monotype full ceramic implant made of zirconiumdioxide (zirconia, ZrO_2_) with a micro-rough surface topography (Zirconia Large-grit sandblasted and Acid-etched, ZLA^®^ surface, Straumann^®^ PURE Ceramic Implant, Institut Straumann AG, Basel, Switzerland, Figure 5). This product has been officially commercially available since April 2014.

For the detailed planning of the implant-supported fixed prosthetic reconstruction, a modified backward planning was used. The prosthetic planning was limited by the fact that, at that time, only implants with a diameter of 4.1 mm were commercially available. After making a diagnostic wax up, it was decided to place three implants: two implants in region 11 and 13 supporting a Fixed Dental Prosthesis (FDP); and one implant in region 14 supporting a single crown.

**Figure 6:**
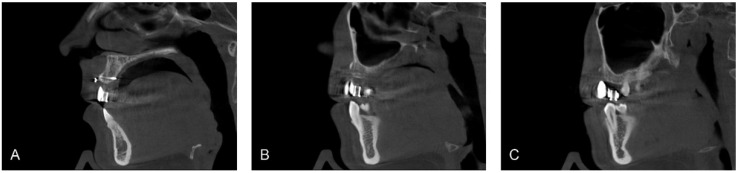
Cone-beam CT-Scan with radiographic stent *in situ* at six months after bone augmentation. Sagittal CT-images representing region 11 (**A**); 13 (**B**) and 14 (**C**). Sufficient vertical and horizontal bone volume in region 11 and 13 (**A**,**B**), reduced horizontal bone volume in region 14 (**C**). Images: Institute of Radiology, Kantonsspital Aarau, AG, Aarau, Switzerland.

After defining the prosthetically optimal implant positions, the dental technician made a radiographic splint containing radiopaque barium teeth. The patient was told to wear the splint during the radiographic investigation. Following this, a new cone-beam CT (Carestream CS9300, Carestream Health, Inc., Rochester, NY, USA; DLP (mGycm): 11.23) was performed. The cone-beam CT scan showed that the bone conditions allowed placing the implants in a prosthetically acceptable position; however, implant placement in region of tooth 14 had to be combined with a simultaneous sinus floor elevation (Figure 6).

**Figure 7:**
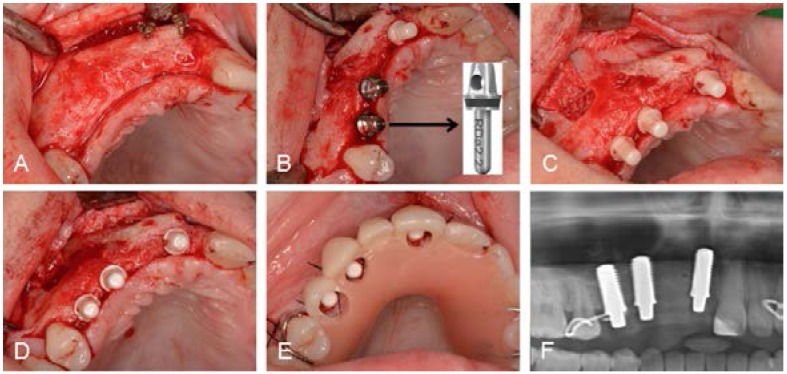
(**A**) Clinical situation after elevation of mucoperiostal flap; (**B**) control of implant positions with implant position indicators; (**C**,**D**) final implant positions, implant in region of 14 was placed in combination with a simultaneous sinus floor elevation; (**E**) adapted occlusally stabilized temporary porsthesis; and (**F**) panoramic radiographic investigation immediately after implant placement. Surgeon: Dr. Stefan Roehling.

In August 2013, three monotype zirconia implants (diameter 4.1 mm, length 10 mm, abutment height 4 mm) were placed in local anesthesia. After elevating a muco-periostal flap and after removing the osteosynthesis screws, the implants could be placed as previously planned in region 11, 13 and 14. At implant placement, the horizontal bone level was sufficient and implants could horizontally be placed in an optimal position. Vertically, the bone volume still showed some deficiencies on the palatal side. Thus, implant placement was limited by compromised anatomical conditions. However, crestal dehiscences could be avoided. During surgery, special implant position indicators were used to check if the implants could be positioned in a prosthetically acceptable position and parallel to each other (Figure 7). Implant placement in region 14 was combined with a sinus floor elevation. For that purpose, autogenous bone chips were collected from the lateral wall of the sinus maxillaris using a cortical bone collector (Safescraper^®^ Twist, Imtegra OHG, Rostock, Germany). Following this, a bony window was prepared with Piezosurgery (mectron s.p.a., Carasco, Italy) to get access to the sinus. After elevation of the sinus membrane, the implant was placed, the previously collected autogenous bone chips were added and the bone window was covered with a collagen membrane (Bio Gide^®^, Geistlich Biomaterials, Baden-Baden, Germany). After suturing, the removable temporary prosthesis that was occlusally stabilized on the neighboring teeth was adapted to the new clinical situation (Figure 7). Ten days after surgery, the sutures were removed.

In December 2013, after four months of osseous integration, the implant shoulders were surgically uncovered in local anesthesia and a dental impression using impression caps (Institut Straumann AG, Basel, Switzerland) was taken. One week later, two temporary prostheses (FDP cemented on implants in region 11 and 13, single crown cemented on implant in region 14) manufactured by the dental technician were temporarily cemented (TempBond, KerrHawe SA, Bioggio, Switzerland) to form the peri-implant soft tissues. After that, follow-up investigations were regularly performed every 2–4 weeks to check the forming of the peri-implant soft tissues.

**Figure 8:**
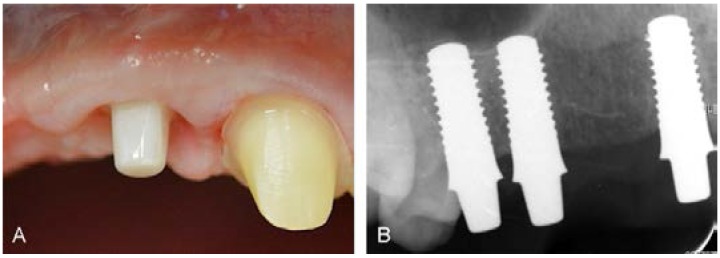
(**A**) Completely non-irritated peri-implant soft tissues of implant in region of 11 just prior to cementation of definitive prosthesis. (**B**) Radiographic investigation at nine months after implant placement, just prior to definitive cementation.

After conditioning of the peri-implant soft tissues (Figure 8A), in June 2014, a new dental impression was taken as previously described. Following this, the definitive prostheses were fixated. Ten months after implant placement and simultaneously performed sinus floor elevation, the radiographic investigation just prior to cementation of the definitive prostheses showed a successful bone remodeling around the implant in region 14 (Figure 8B). The definitive fixation of the suprastructures was done according to a defined cementation protocol, which included a moderate/thin application of the cement (glass ionomer cement) by using a small dental brush. Additionally, during the hardening procedure, cement excess was removed with a special dental floss (Superfloss, Procter & Gamble GmbH, Schwalbach am Taunus, Germany). Additionally, in the same session a full ceramic veneer was cemented on tooth 21 with luting agent (Panavia^TM^, Kuraray Europe GmbH, Hattersheim am Main, Germany).

**Figure 9:**
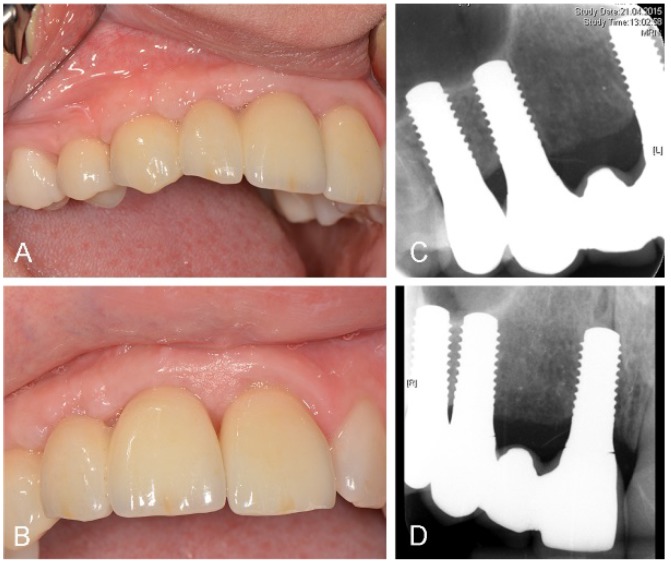
Clinical (**A**,**B**) and radiographic (**C**,**D**) investigation at 20 months after implant placement. FDP cemented on implants in region 11 and 13, single crown cemented on implant in region 14. Prosthetic reconstruction: Dr. Thomas Borer.

The latest follow-up investigation at 20 months after implant placement in April 2015 showed clinically and radiographically completely non-irritated peri-implant soft and bone tissue conditions (Figure 9).

## 3. Discussion

In the present case, new monotype full ceramic implants of the latest generation have been used. The implants were made of zirconiumdioxide and had a micro-rough surface topography (ZLA™ surface, Straumann^®^ PURE Ceramic Implant, Institut Straumann AG, Basel, Switzerland), which is similar to the well-known SLA surface (Sandblasted with Large Grits and Acid etched, [2]).

Initially, a lot of inconsistent results have been reported with regard to the clinical performance of zirconia dental implants. In the literature, many different kinds of zirconia implants have been investigated and the reported survival rates (range between 0% and 98%) as well as the investigation periods (range between 8.2 and 60 months) range widely [32]. Thus, a reliable clinical interpretation of these results is hardly possible. Consequently, a recently published literature review concluded—based on studies that were published between 2006 and 2011—that zirconia implants are inferior to titanium implants with regard to survival and success rates [32]. Interestingly, 16 out of the 17 studies that were included in the latter review investigated monotype implant systems, and only one case report dealt with a two-part system [32]. However, it must be noticed that the latter review only included “first-generation” zirconia dental implants with a rather smooth surface topography.

Zirconia dental implants of the latest generation with a micro-rough surface topography show similar survival and success rates compared to established data on moderately rough titanium implants. In detail, monotypes as well as two-part implant systems have been investigated and the reported survival rates ranged between 93.3% and 98.2% for investigation periods between one and five years [33,34,35,36,37,38,39,40]. Only one recently published study reported survival rates of only 87% and 86% after one and two years of follow-up, respectively [41]. Interestingly, the authors of the latter study related the zirconia implant failures to an “aseptic loosening” and not to peri-implant infections. Most recently, a multicenter study has been published that investigated the same type of zirconia implants that were used in the present case. Forty-four patients with single tooth gaps received a total of 44 zirconia implants with a ZLA® surface topography. Twelve months after implant placement, the zirconia implants showed a survival and success rate of 97.6%. Additionally, the authors reported mean bone loss of 0.88 mm between implant placement and implant loading and mean bone loss of 0.14 mm between implant loading and 12 months after placement [33]. Thus, from a clinical point of view, zirconia implants of the latest generation—like those used in the present case—seem to be a reliable alternative to moderately rough titanium implants. However, further prospective studies have to confirm the clinical long-term reliability of micro-roughened zirconia implants.

In the present case, six months prior to implant placement, a vertical bone augmentation with an autogenous bone block harvested from the mandibular ramus was performed. In addition to that, implant placement in region 14 was combined with a simultaneous sinus floor elevation using autogenous bone chips. The clinical and radiographic investigations clearly showed that—in the present case—these new zirconia implants could be successfully combined with previously or simultaneously performed autogenous bone augmentation procedures. Evidence-based investigations have reported survival rates between 96.9% and 100% for titanium implants placed after autogenous block augmentations and a mean survival rate of 97.4% ± 2.2% for implants placed after maxillary sinus floor augmentations using autogenous bone [42,43]. Within the limits of a case study, it seems that the presently used surgical techniques can be considered as reliable and well-accepted treatment procedures that can also be used with micro-roughened zirconia dental implants.

Prior to implant placement, a vertical bone augmentation was only performed at the labial side of the maxilla. Thus, six months later the local vertical bone volume was sufficient but still compromised especially on the palatal side of the alveolar process. Consequently, implant placement was limited by compromised bone conditions. When using monotype implants, it is very important to place the implants prosthetically driven (*i.e.*, correct implant position and axis) since it is not possible to make major corrections of the implant axis by using individual abutments. However, the present case shows that even in compromised bone conditions monotype zirconia implants can be used. All implants were placed in a prosthetically acceptable position, aligned parallel to each other resulting in a functionally and esthetically highly satisfying fixed prosthetic reconstruction.

Due to the monotype design of the implants, the abutment penetrates into the oral cavity directly after implant placement. Thus, the avoidance of premature implant overloading might be a limiting factor during the osseointegration period. In the present case, the temporary prosthesis was occlusally stabilized on the neighboring teeth and directly after surgery, the prosthesis was adapted to the new clinical situation. Thus, implant overloading could successfully be avoided.

In the literature, non-loaded healing periods between 4.1 and 4.3 months after implant placement combined with simultaneous sinus floor augmentations with autogenous bone have been reported for moderately rough titanium implants [43,44]. Since many experimental studies have shown no significant differences between the presently used micro-rough zirconia implant surface compared to the well-known Titanium-SLA (Ti-SLA) surface [28,29,30,31], an identical protocol for the healing period was used. Thus, after implant placement and simultaneous sinus floor elevation, all implants were functionally loaded after four months of non-loaded healing. It should the noticed that the reported experimental data was evaluated within the limits of preclinical animal studies [28,29,30,31]. However, the published evidence based data [28,29,30,31,33] and the successful treatment in the present case is very promising with regard to the clinical application of micro-rough zirconia implants.

Clinically at 20 months after implant placement, completely non-irritated peri-implant soft tissue conditions and naturally shaped papilla formation has been observed. Identical observations were reported for a different type of monotype zirconia implants after a functional loading period of up to and after seven years [45,46]. This excellent soft tissue attachment might be related to the monotype character of the used implant avoiding subgingival micro-gaps and to the material properties of zirconia showing superior biocompatibility compared to metals. In detail, a statistically significantly increased microcirculation in the peri-implant soft tissues has clinically been shown for zirconia in comparison to titanium [47]. In addition to that, different studies have even reported a statistically significantly reduced bacterial adhesion on zirconia surfaces in comparison with titanium surfaces [48,49,50] and also less inflammatory cells in the peri-implant soft tissue of zirconia in comparison with titanium or other metals [51,52].

## 4. Conclusions

The present case demonstrated a successful treatment using zirconia dental implants for the reconstruction of a large edentulous area with compromised local bone conditions resulting in highly satisfying functional and esthetical outcomes. In addition to that, the clinical and radiographic investigations have clearly shown that—in the present case—zirconia implants with a micro-rough surface topography could successfully be combined with previously or simultaneously performed autogenous bone augmentation procedures. However, further well-designed prospective clinical studies have to be conducted to investigate the clinical long-term performance of zirconia dental implants.
